# Supplementary material for: Genome-Wide Identification and Functional Analysis of the GASA Gene Family Responding to Multiple Stressors in Canavalia rosea
Source: Genes (Basel). 2022 Oct 31;13(11):1988. doi: 10.3390/genes13111988 (PMC9690345; doi:10.3390/genes13111988)
Supplement: Supplementary file 1 [file genes-13-01988-s001.zip › genes-1909889-supplementary.pdf]

genes

MDPI

Supplementary Material Information

Genome-Wide Identification and Functional Analysis of the *GASA* Gene Family Responding to Multiple Stressors in *Canavalia rosea*

Mei Zhang [1,2,*], Zhengfeng Wang [1,3,4] and Shuguang Jian [1,3,*]

[1] Guangdong Provincial Key Laboratory of Applied Botany, South China Botanical Garden, Chinese Academy of Sciences, Guangzhou 510650, China
[2] Key Laboratory of South China Agricultural Plant Molecular Analysis and Genetic Improvement, South China Botanical Garden, Chinese Academy of Sciences, Guangzhou 510650, China
[3] Key Laboratory of Vegetation Restoration and Management of Degraded Ecosystems, South China Botanical Garden, Chinese Academy of Sciences, Guangzhou 510650, China
[4] Key Laboratory of Carbon Sequestration in Terrestrial Ecosystem, South China Botanical Garden, Chinese Academy of Sciences, Guangzhou 510650, China
* Correspondence: zhangmei@scbg.ac.cn (M.Z.); jiansg@scbg.ac.cn (S.J.)

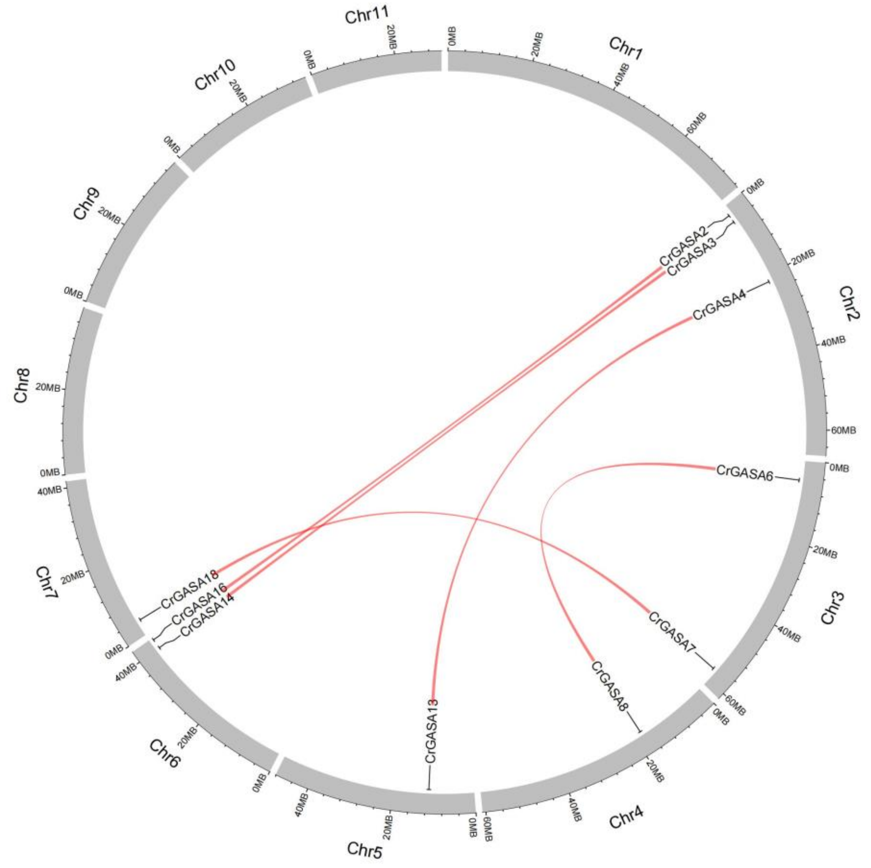

Figure S1. The distribution of segmental duplication of *CrGASA*s in *Canavalia rosea* chromosomes.

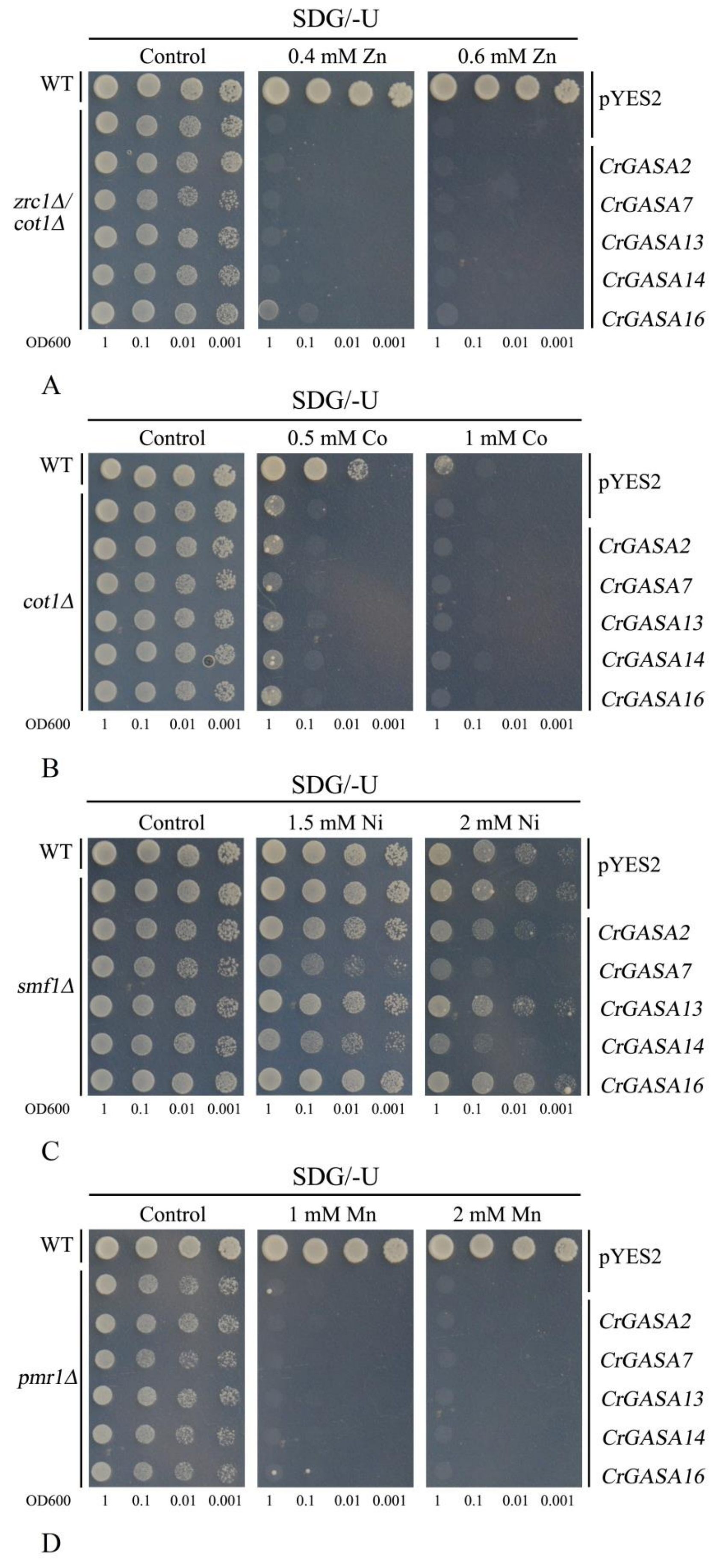
SDG/-U
Control
0.4 mM Zn
0.6 mM Zn
WT
zrc1Δ/
cot1Δ
pYES2
CrGASA2
CrGASA7
CrGASA13
CrGASA14
CrGASA16
OD600
1 0.1 0.01 0.001
1 0.1 0.01 0.001
1 0.1 0.01 0.001
A
SDG/-U
Control
0.5 mM Co
1 mM Co
WT
cot1Δ
pYES2
CrGASA2
CrGASA7
CrGASA13
CrGASA14
CrGASA16
OD600
1 0.1 0.01 0.001
1 0.1 0.01 0.001
1 0.1 0.01 0.001
B
SDG/-U
Control
1.5 mM Ni
2 mM Ni
WT
smf1Δ
pYES2
CrGASA2
CrGASA7
CrGASA13
CrGASA14
CrGASA16
OD600
1 0.1 0.01 0.001
1 0.1 0.01 0.001
1 0.1 0.01 0.001
C
SDG/-U
Control
1 mM Mn
2 mM Mn
WT
pmr1Δ
pYES2
CrGASA2
CrGASA7
CrGASA13
CrGASA14
CrGASA16
OD600
1 0.1 0.01 0.001
1 0.1 0.01 0.001
1 0.1 0.01 0.001
D

Figure S2. Complementation confirmation of yeast mutants mediated by five *CrGASA*s on solid medium containing heavy metals (HMs). (A) Zn (*zrc1Δ/cot1Δ*); (B) Co (*cot1Δ*); (C) Ni (*smf1Δ*); and (D) Mn (*pmr1Δ*). The WT strain BY4741 was transformed with the empty vector pYES2, and the mutant strains were transformed with pYES2 or with recombinant vectors CrGASAs-pYES2. Yeast cultures were adjusted to OD600 =1, and 2 μL of serial dilutions (10 fold, from left to right in each panel) were spotted on SDG/-Ura medium supplemented with different concentrations of HMs. The corresponding yeast spots growing on SDG/-Ura plates without HMs were used as controls. The plates were incubated for 2–5 days at 30°C.

Table S1. Primer sequences used in this study.

Table S2. The sequences of *CrGASA* genomic DNA, CDS, and promoter region DNA.

Table S3. Summary of possible candidate *cis*-regulatory elements found in *CrGASA* promoter DNA regions.

Table S4. The FPKM values of *CrGASA*s for RNA-Seq assay of *C. rosea* tissues in this study.
